# Molecular Characterization of *MCR-1* Producing *Enterobacterales* Isolated in Poultry Farms in the United Arab Emirates

**DOI:** 10.3390/antibiotics11030305

**Published:** 2022-02-24

**Authors:** Ágnes Sonnevend, Walid Q. Alali, Sara A. Mahmoud, Akela Ghazawi, Greeshma Bharathan, Szilvia Melegh, Tahir A. Rizvi, Tibor Pál

**Affiliations:** 1 Department of Medical Microbiology and Immunology, Medical School, University of Pécs, 7624 Pécs, Hungary; pal.agnes@pte.hu (Á.S.); melegh.szilvia@pte.hu (S.M.); 2 Department of Medical Microbiology and Immunology, College of Medicine and Health Sciences, United Arab Emirates University, Al Ain 15551, United Arab Emirates; saraamahmoud100@yahoo.com (S.A.M.); akelag@uaeu.ac.ae (A.G.); greeshmabharathan@gmail.com (G.B.); tarizvi@uaeu.ac.ae (T.A.R.); 3 Department of Epidemiology and Biostatistics, Faculty of Public Health, Kuwait University, Safat, Kuwait City 13110, Kuwait; walidalali@gmail.com; 4 Department of Food Science, College of Agriculture and Veterinary Medicine, United Arab Emirates University, Al Ain 15551, United Arab Emirates; 5 Zayed Center for Health Sciences, United Arab Emirates University, Al Ain 15551, United Arab Emirates

**Keywords:** mobile colistin resistance, poultry, multi-drug resistance, Middle East

## Abstract

Data on the prevalence of MCR-producing *Enterobacterales* of animal origin are scarce from the Arabian Peninsula. We investigated the presence and variety of such strains from fecal specimens of poultry collected in four farms in the United Arab Emirates. Colonies from ten composite samples per farm grown on colistin-supplemented plates were PCR-screened for alleles of the *mcr* gene. Thirty-nine isolates selected based on species, colony morphology, and plasmid profile were subjected to whole genome sequencing. The panel of their resistance and virulence genes, MLST and cgMLST were established. Transferability and incompatibility types of the MCR-plasmids were determined. *mcr-1.1* positive strains were identified in 36 of the 40 samples. Thirty-four multi-drug resistant *Escherichia coli* of 16 different sequence types, two *Escherichia albertii*, two *Klebsiella pneumoniae* and one *Salmonella minnesota* were identified. Beyond various aminoglycoside, tetracycline, and co-trimoxazole resistance genes, seven of them also carried ESBL genes and one *bla*_CMY-2_. Six IncHI2, 26 IncI2 and 4 IncX4 MCR-plasmids were mobilized, in case of the IncHI2 plasmids co-transferring ampicillin, chloramphenicol and tetracycline resistance. The diversity of *mcr-1* positive strains suggest a complex local epidemiology calling for a coordinated surveillance including animals, retail meat and clinical cases.

## 1. Introduction

Albeit human use of polymyxins had been considerably reduced in the 70s and 80s due to their toxicity, they have been continuously applied in veterinary medicine and in animal husbandry in several developing and developed countries [1,2]. Lately, the emergence of multi-drug resistant (MDR) Gram-negative bacteria necessitated their reintroduction into human medicine, which was followed by increasing rate of colistin resistance. This alarming phenomenon has been best studied among carbapenem resistant Gram-negative rods, i.e., the primary targets of polymyxin use [3,4]. Recently, this already distressing scenario was further aggravated by the description of mostly plasmid, and sometimes chromosomally located mobile colistin resistance genes (*mcr*) [5,6].

While human cases have been recorded globally, the majority of isolates carrying *mcr* genes were isolated from food, or from food-producing animals, raising the possibility that human infections may, at least partly, originate from these sources [5].

Carbapenem resistant *Enterobacterales* (CRE) infections have been a major problem in countries of the Arabian Peninsula [7,8], with colistin resistance rate of >20% reported in certain CRE collections [9,10]. Although various *mcr* genes have been documented among sporadic human isolates [11,12,13,14,15,16,17,18], other studies did not find them at all [9], suggesting that colistin resistance of clinical isolates in the region is mainly due to chromosomal mutations [10]. While the presence and variety of *mcr*-carrying strains in poultry has been extensively documented in Egypt, Lebanon and Pakistan [19,20,21,22,23,24], the only similar study in the Arabian Peninsula described 14 *mcr*-carrying *Escherichia coli* from Qatar without any further characterization [25]. The aim of the current study was to investigate whether *mcr*-carrying *Enterobacterales* strains are present in faecal samples of healthy broiler chicken in four randomly selected farms in the Eastern region of Abu Dhabi Emirate (United Arab Emirates) and if yes, to assess their clonal diversity, as well as the variety of the *mcr-*containing mobile genetic elements carried. 

## 2. Results

### 2.1. Identification and Antibiotic Susceptibility of the mcr-1 Carrying Strains

Thirty six of the 40 composite samples collected (90%) yielded colonies positive for the *mcr-1* gene, i.e., multiple samples from each of the four farms contained such strains. All together 216 *mcr* positive isolates were identified. Based on the species, colony morphology and unique plasmid profiles, a total of 39 strains were selected for further studies (Supplementary Table S1).

These strains were subjected to whole genome sequencing (WGS), which confirmed the identification of 34 *E. coli*, two *Escherichia albertii*, two *Klebsiella pneumoniae* and 1 *Salmonella enterica* subspecies Enterica strain predicted in slico to be serovar Minnesota (Supplementary Table S1). 

All 39 isolates were resistant to colistin with MIC ranging 4->256 mg/L, and all but one strain qualified as multi-drug resistant. (Supplementary Table S2). 

The array of acquired resistance genes identified by ResFinder matched the phenotypic susceptibility results. All strains possessed the *mcr-1.1* allele, and one or more beta-lactamase genes. The eight isolates resistant to 3rd generation cephalosporins carried either an extended spectrum beta-lactamase (ESBL) (*bla*_CTX-M-15_, *bla*_SHV-12_) or an *ampC* gene (*bla*_CMY-2_), respectively. Furthermore, the isolates possessed a variety of acquired aminoglycoside, macrolide, phenicol, tetracycline, trimethoprim, sulphonamide, quinolone, rifampicin and fosfomycin resistance genes (Supplementary Table S2). The two *K. pneumoniae* strains exhibiting colistin MIC > 256 mg/L also carried mutations in their *pmrB* gene T246A and R256G. 

### 2.2. Results of the Molecular Typing

**Figure 1:**
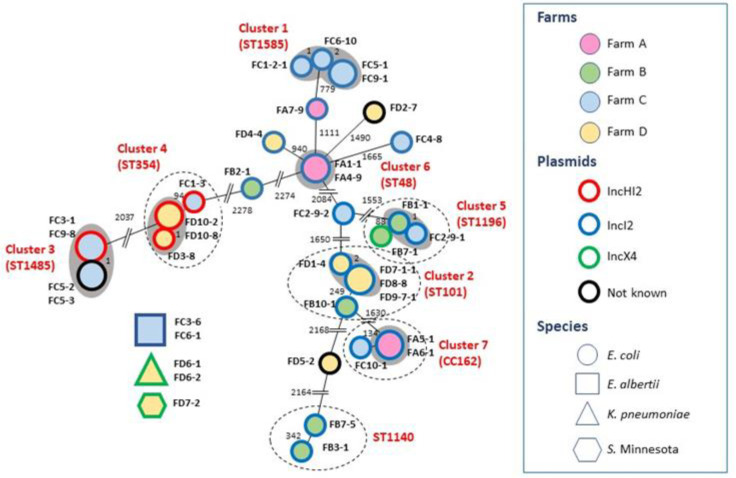
Minimum spanning tree based on the cgMLST of the mcr-carrying *Escherichia coli* isolates (numbers on branches represent the differences in the 2513 alleles examined; grey halo marks clusters, i.e., isolates with ≤10 alleles differences; isolates circled with dotted lines belong to the same sequence type or clonal complex even if outside of a particular cgMLST cluster).

Multi-locus sequence typing (MLST) and core genome MLST analysis was performed for the two *K. pneumoniae* and the 34 *E. coli* isolates. The *K. pneumoniae* strains belonged to ST340 and had identical cgMLST types. The 34 *E. coli* belonged to 16 sequence types. Eight *E. coli* STs were represented by multiple isolates. Three of them (ST101, ST354 and ST1196, respectively) were encountered at multiple farms. Strains clustering by cgMLST were present in seven MLST types. Except for Cluster 5, all cgMLST clusters contained strains from the same farm. Further 13 *E. coli* strains did not cluster by cgMLST. (Supplementary Table S1 and Figure 1). The two *E. albertii*, isolated from the same farm, exhibited identical PFGE patterns.

### 2.3. Virulence Genes of the mcr-Carrying Isolates

Of the 34 *E. coli* strains, 24 had more than ten of the 40 virulence related genes identified (Supplementary Table S3), and the average number of such genes per strain in the collection was 12.09. It was noteworthy that STs represented by multiple (≥3) strains (i.e., ST101, ST1196, ST1485, ST1585 and ST354, respectively) carried more virulence factor genes (14.65 ± 4.58) than strains representing sporadic isolates or smaller groups (8.43 ± 5.09) (*p* < 0.001). The most common genes carried by over 50% of the isolates were lpfA, hlyF, iroN, iucC, iutA, sitA, iss, traT, ompT, terC. 

The two *E. albertii* carried eae, cdtB, traT and terC genes. The *S. minnesota* strain had colicin ib (cib), yersiniabactin (fyuA and irp2) and serum resistance (traT) genes. Similarly, the two *K. pneumoniae* isolate possessed colicin ib gene (cib), several siderophore (iutA, fyuA and irp2) and serum resistance (traT) genes.

### 2.4. Characterization of the MCR Plasmids

*mcr-1* bearing plasmids were transferable from 26 of the 39 wild-type isolates. Conjugation resulted in single plasmid-containing transconjugants in case of 20 isolates, while in six conjugations the transconjugants acquired more than one plasmid. From the latter ones, subsequent transformation resulted in single mcr-plasmid derivatives. From further nine wild type strains direct transformation, but not conjugation resulted in single mcr-plasmid derivatives. Based on the comparison to plasmid size control (*E. coli* 39R861), the mcr-plasmids were approx. 35, 60 or >150 kb in size. Of the four wild type isolates, from which no single plasmid derivatives were obtained, attempts were made to localize the mcr-plasmids by hybridization. In one of them (FD2-7) the probe marked a >150 kb plasmid (data not shown). In the remaining three isolates the plasmid localization of the mcr-1 could not be established (Supplementary Table S1).

Single plasmid derivatives were subjected to RFLP analysis. It was successful with all but two plasmids (pFB7-1-mcr and pFD7-2-mcr). In case of the 60 kb plasmids two, of the ~35 kb plasmids a single, and of the >150 kb plasmids three RFLP types were identified, respectively (Supplementary Table S1). At least one representative of each subtype and the two plasmids for which no clear RFLP patterns could be obtained were subjected to plasmid sequencing. 

All 60 kb plasmids proved to be IncI2 types. The >150 kb plasmids exhibited IncHI2, while the 35 kb belonged to IncX4 incompatibility types, respectively. The plasmids not sequenced but having the same size and exhibiting the same RFLP patterns as the one sequenced were considered as belonging to the same type, as indicated in Supplementary Table S1.

**Figure 2:**
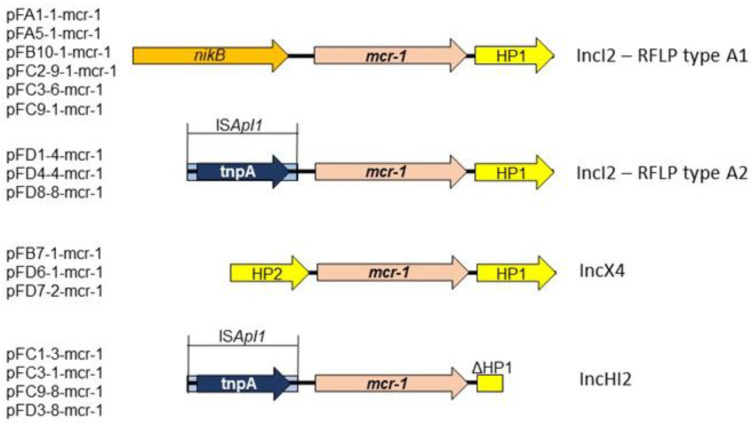
Genetic surrounding of the *mcr-1* gene in various plasmids.

The immediate surrounding of the *mcr-1* genes varied according to the Inc types of the plasmids. Among the IncI2 plasmids two distinct structures were identified correlating to RFLP types A1 and A2, respectively, as shown in Figure 2. In the IncI2-RFLP type A2 and IncHI2 plasmids the mobile element ISApl1 was found upstream of the gene.

Members of a single *E. coli* ST (ST1196) carried plasmids of two different incompatibility types, i.e., IncI2 (strain FC2-9-1) or IncX4 (strain (FB7-1), only. Beyond *E. coli* FB7-1, the IncX4 plasmid was also present in the two *K. pneumoniae* and in the *S. minnesota* strains (Supplementary Table S1).

*E. coli* MLST clones with multiple members usually carried an IncI2 plasmid. With the exception of ST101, within the same ST the RFLP types of the plasmids were identical. On the other hand, while 4 members of ST101 isolated from farm D carried RFLP pattern A2 of the 60 kb IncI2 plasmid and formed a cgMLST cluster (Cluster 2, Figure S1), the single isolate of the same ST recovered from farm B harboured the RFLP pattern A1 version of the plasmid and did not cluster by cgMLST (Supplementary Table S1, Figure 1). 

**Figure 3:**
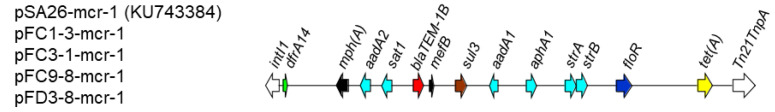
The resistance island of the IncHI2 plasmids of this study and that of pSA26-mcr-1 (of a human isolates described in [14]).

Transfer of all mcr-plasmids resulted in a considerable increase of colistin MIC of the recipients. While transfer of the IncI2 and IncX4 plasmids did not, that of the IncHI2 plasmids resulted in co-transfer of resistance to multiple antibiotics (Supplementary Table S1). The resistance island of these plasmids contained the bla_TEM-1B_, dfrA14, sul3, tet(A), mph(A), floR, aadA1, aadA2, sat1, aphA, strA and strB genes, i.e., a structure identical to one described earlier in a plasmid (pSA26-*mcr-1*) from a human isolate from Saudi Arabia [14] (Figure 3).

### 2.5. Variety of the Wild Type Strains Encountered

Based on the species, in case of *E. coli* the different STs, and the MCR-plasmids carried, among the 39 *mcr-1* positive strains investigated in details 19 different variations have been encountered. The most homogenous population was found in Farm A (3 types), the most heterogenous was encountered in Farm C (8 types) (Table 1). 

## 3. Discussion

Resistance to colistin has been extensively documented in countries of the Arabian Peninsula [8,9,10]. Although *mcr*-mediated colistin resistance among clinical isolates have been encountered in almost all countries of the region, these were usually sporadic MDR, often carbapenem resistant strains [10,11,12,13,14,15,16,17,18] and the colistin resistance among the majority of them seemed to be chromosomally encoded [9,10]. Contrary of the paucity of *mcr* harboring clinical isolates, we found that fecal specimens of broiler poultry contained a surprising variety of MCR-producing colistin resistant strains (*E. coli* of 16 different STs, *E. albertii*, *K. pneumoniae* and *S. minnesota*), matching or exceeding the results of similar studies from countries of the extended neighborhood [19,20,21,22,23,24]. To the best of our knowledge, with the exception of ST1585, all STs identified in this study have been described to carry the *mcr* gene [2]. Furthermore, several of the STs encountered, such as ST101 [22,23], ST1011, ST1140, ST93 [20] ST1196 [20,22], ST48 [20,23] have even been identified among *mcr-1* carrying strains recovered from poultry or chicken meat in countries close to the region of the current study. Although *Salmonella enterica* strains of animal origin have been frequently associated with different alleles of *mcr* [26], to the best of our knowledge, our study is the first identifying *S. minnesota* carrying *mcr-1*. Worryingly, this strain, beyond resistant to six other classes of antibiotics, due to the presence of *bla*_CMY-2_, also exhibited resistance to extended spectrum cephalosporins, a feature causing recent concerns globally [27]. *E. albertii*, a well-known human and animal pathogen, has previously been described to carry the *mcr-1* gene but only in China, and not in the Middle-East [28]. It was noteworthy that the two *K. pneumoniae* isolates exhibiting colistin MIC >256 mg/L also carried mutations in their chromosomally encoded PmrB, at least one of which (R256G) were previously described as deleterious [29] 

It is commonly accepted that food producing animals are important sources of *mcr* carrying strains [1]. This can, at least partly be explained by the fact that avian pathogenic *E. coli* (APEC) often share some of the STs [30] and virulence factors with human extraintestinal pathogenic *E. coli* (ExPEC) [31]. In this study we also encountered such STs (ST48, ST93) [30] and several of the virulence factors present in over 50% of our strains (e.g., *hlyF, iroN, iutA, sitA, traT,* Supplementary Table S3) are also commonly found in both APEC and ExPEC isolates [31]. 

Further to their potential disease-causing capacity, these stool isolates carried several resistance genes beyond *mcr-1.* Although none of the strains were carbapenem resistant, all were phenotypically multi-drug resistant. Most of them had TEM-1 beta-lactamase, sulfonamide, trimethoprim, tetracycline resistance and aminoglycoside modifying enzyme coding genes (Supplementary Table S2). Worryingly, eight of the 39 strains carried genes coding for to 3rd generation cephalosporin resistance (*bla*_CTX-M-15_, *bla*_SHV-12_, *bla*_CMY-2_), one third of them had plasmid mediated fluoroquinolone resistance *qnr* gene, two had 16S-methylase *rmtB* coding for resistance to all aminoglycosides. Furthermore, an ESBL producer *E. coli* (FA7-9) also carried a *fosA3* and was phenotypically fosfomycin resistant. Such multidrug resistant isolates have also been increasingly noted among *mcr-1* carrying strains in the region and world-wide [5,21,22,23,32,33].

In 35 of the 39 strains the *mcr-1* gene could be localized on plasmids, of which 26 could be transferred by conjugation either as a single plasmid or together with other episomes (Supplementary Table S1). Contrary to the diversity of species and sequence types of MCR expressing isolates encountered, *mcr*-carrying plasmids identified in this study belonged to only three Inc types, i.e., IncX4, IncI2 and IncHI2. This relative paucity of the plasmid scaffolds compared to the variation of the diversity of clones is in agreement with global observations [2,5], as well as with those from other Middle Eastern countries outside of the Arabian Peninsula [13,14,16,18,20,22]. With the exception of *E. coli* ST1196 carrying either IncI2 or IncX4 plasmids, the various types of plasmids were associated with specific clones. On the other hand, beyond *E. coli*, IncI2 plasmids were present in *E. albertii* and IncX4 MCR plasmids in *K. pneumoniae* and *S. minnesota* (Supplementary Table S1). These findings are also in line with other observations, noting that IncI2 and IncX4 plasmids are two of the most common plasmids known to transfer *mcr-1* among various species of the *Enterobacterales* [2,34]. 

Only the large, IncHI2 plasmids carried transferable antibiotic resistance genes besides *mcr-1*. A notable difference between the IncHI2 plasmids of our study and the same incompatibility type *mcr*-plasmids of human isolates from the Arabian Peninsula [12,14] was noted in the immediate genetic surrounding of the *mcr-1* gene. In the poultry isolates the IS*Apl1* did not bracket the *mcr-1* gene, it was found only upstream of it. Nevertheless, the IncHI2 plasmids of chicken origin harbored the same resistance island of 13 resistance genes, as a human isolate of Saudi Arabia (Figure 3), suggesting possible transfer between animals and humans [14].

The study has some important limitations. First, it did not reveal the actual frequency neither of the colistin resistant strains, nor of *mcr* positive ones in the farms investigated. Nevertheless, as 90% of the pooled samples were positive for such isolates, we assume that both rates can be considerable. The selection of *mcr* carrying strains for WGS could have introduced some bias limiting the variety of strains encountered. Nevertheless, we feel that since all isolates representing unique colony morphology and plasmid profile combinations from each farm were further studied, the impact of this pre-selection limitation could be relatively low. The limited panel of *mcr* alleles (i.e., *mcr* 1-5) the strains were initially screened for leaves the possibility open that isolates carrying further alleles were missed by our study while they would further extend the variability of colistin resistant strains present. Furthermore, the low number of farms and samples studied does not support any generalization of our findings to other broiler farms in the country or in the larger region. However, the farms were randomly selected, and we have no indication that they are different from others in any possible aspects. The study did not specifically address either whether any transfer of *mcr*-carrying strains has been actually taking place between the farms. The fact that all plasmid Inc types were present in multiple farms does not prove inter-farm transfer *per se*, as these three types are the most wide-spread *mcr*-carrying plasmid scaffolds globally, as well as in the Middle East [2,5,22]. Whether local transmission of these plasmids between strains, or even species has contributed to the complex picture encountered, or strains carrying identical plasmids where independently introduced to the local farms should be the subject to further, more detailed investigations.

The fact that with the exception of one (cluster 5), all cgMLST clusters exclusively contained isolates recovered from the same farm suggests that inter-farm clonal spread may not be the dominating mechanism behind the wide-spread presence of *mcr* 1.1. carrying isolates. On the other hand, the existence of such mechanism cannot completely be ruled out either, as in multiple cases isolates from farms different from those in clusters mapped close and shared the same MLST (clusters 2, 4, 5 and 7, respectively, Figure 1). 

A question yet to be answered is to what extent *mcr*-carrying strains in the farms of the region enter the food-chain and colonize and infect human hosts. No such studies have been conducted so far in the countries of the Peninsula, while the presence of such strains have been documented in retail meat or samples collected in slaughterhouses in other Middle Eastern countries [20,22]. The majority of reports on human MCR expressing strains focusing on sporadic CRE is unlikely to reveal the entire picture. Albeit often MDR, most of the isolates from poultry are still susceptible to carbapenems, i.e., type of isolates seldom tested for colistin resistance in clinical laboratories. Recent observation [35,36] on the high rate of colonization of pilgrims returning from Hajj with *mcr*-positive, but not necessarily carbapenem resistant strains suggests that indeed, such exposure in countries of the Peninsula is likely and our data provide some evidence that these strains do infest flocks of local broilers, i.e., a potential source for such isolates.

The considerable diversity of strains encountered even in this small-scale study (Table 1) suggests a complex local epidemiology of *mcr*-carrying strains with likely multiple sources of introduction to the farms. We believe that these findings warrant extended, country-wide investigations involving strains isolated from animals, retail meat and clinial cases to reveal the dynamics of possible transfer of resistant strains and resistance genes between different niches in the region.

## 4. Materials and Methods

### 4.1. Sample Collection

After approaching owners of 8 broiler poultry farms in the Eastern region of Abu Dhabi Emirate, United Arab Emirates (UAE) four agreed to the study. These four farms, privately owned by different independent owners, and located 15 to 70 km from the city of Al Ain and 30 to 90 km from each other were visited between March-April 2018 for sample collection. Each farm was composed of 2 to 4 broiler houses. The number of broilers in each house ranged between 5000 and 7000 birds. All were healthy, i.e., no outbreaks of infectious diseases occurred in the broiler flocks during the sample collection. During each visit, one randomly selected broiler house was sampled. Birds at the time of visits were 3-weeks of age. From each of the four farms ten composite faecal samples (~100 g each) were collected and placed in sterile plastic containers. Each composite sample was composed of three, separate fresh faecal droppings. The sampling was carried out using a zig-zag pattern through the entire broiler house as described earlier [37]. The faecal samples were transferred to the laboratory and processed on the day of collection. 

### 4.2. Detection and Identification of Mobile Colistin Resistance (mcr 1-5) Gene Carrying Isolates

After mechanically mixing, approximately one gram of composite faecal sample was inoculated into 4 mL Tryptic Soy Broth (TSB) (MAST, Merseyside, UK) supplemented with 1 µg/mL colistin sulphate (Sigma-Aldrich, St. Louise, MO, USA) and 8 µg/mL vancomycin (Sigma-Aldrich, St Louise, MO, USA). After overnight incubation at 37 °C, two McConkey agar plates (Oxoid, Basingstoke, UK) containing 1 µg/mL colistin sulphate (Sigma-Aldrich, St Louise, MO, USA) were inoculated from each sample with 10 µL and 100 µL of the TSB culture, and these were incubated overnight at 37 °C. If less than 10 isolated colonies grew, all were subcultured as macrocolonies on Tryptic Soy Agar (MAST, Merseyside, UK) supplemented with 1µg/mL colistin sulphate and Technical Agar N^o^3 (Oxoid, Basingstoke, UK) up to 3% agar content to prevent swarming. In case of >10 isolated colonies per sample were present, ten colonies with different colony morphology were selected for subculture.

Heat extracted DNA of the macrocolonies were tested for the presence of mobile colistin resistance determinants known at the time of investigation by multiplex PCR detecting *mcr-1, mcr-2, mcr-3* and *mcr-4* [38] and *mcr-5* [39]. From each of the 40 faecal samples up to 6 *mcr-*carrying isolates were selected for further analysis, while *mcr* negative colonies were not studied further. If *mcr* positive isolates exhibited different colony morphology, one of each type was selected. Strains selected were stored in TSB (MAST, Merseyside, UK) containing 20% glycerol at −80 °C until further investigation.

The species of the *mcr* positive strains was determined by MALDI-TOF MS analysis (Bruker Biotyper Microflex LT/SH) and ambiguous results were confirmed by sequencing the 16S ribosomal RNA gene [40]. The strains’ plasmid profiles were established by the alkaline lysis method using *E. coli* V517 and *E. coli* 39R861 for plasmid size controls, as described [41]. Plasmid patterns were compared by the Dice similarity index using GelComparII software v6.5 (Bionumerics, Sin Martens, Belgium). From each farm, representative strains of each species and plasmid patterns were selected for further analysis.

### 4.3. Antibiotic Susceptibility Testing

Antibiotic susceptibility of the isolates was tested according to the Clinical Laboratory Standards Institute [42] using *E. coli* ATCC25922 as quality control. Amoxicillin/clavulanate, ampicillin, cefoxitin, cefpodoxime, ceftazidime, cefotaxime, aztreonam, piperacillin/tazobactam, imipenem, ertapenem, meropenem, gentamicin, amikacin, tobramycin, chloramphenicol, doxycycline, tetracycline, ciprofloxacin, nalidixic acid and co-trimoxazole susceptibility was assessed by disc diffusion. Fosfomycin susceptibility was tested by agar dilution using Muller-Hinton Agar (Oxoid, Basingstoke, UK) supplemented with 25 mg/L glucose-6-phosphate. The minimum inhibitory concentration of colistin was determined by broth microdilution (BMD) in cation adjusted Muller-Hinton Broth (Oxoid, Basingstoke, UK) using colistin sulphate (Sigma-Aldrich, St. Louise, MO, USA). For colistin BMD an *mcr-1* positive *E. coli* isolate (ABC149) [14] with colistin MIC of 4 mg/L was also included. Strains were considered multi-drug resistant if exhibiting non-susceptibility to ≥3 different classes of drugs tested.

### 4.4. Molecular Typing of the Strains by Whole Genome Sequence Based MLST and cgMLST

From each farm, representative strains of each species and plasmid patterns were selected for whole genome sequencing (WGS). Genomic DNA was extracted by Wizard^®^ Genomic DNA Purification Kit (Promega, Madison, WI, USA). 150 bp paired-end whole genome sequencing was carried out on Illumina HiSeq platform as a commercial service by Novogene Company Limited, Hong Kong. The reads were assembled into contigs using CLC Genomic Workbench v20.0 (QIAGEN Aarhus, Denmark). The assembly statistics is provided in Supplementary Table S4. The contigs were uploaded to PathogenWatch (https://pathogen.watch) (accessed on 5 December 2021) to confirm species identification. The acquired resistance gene content was assessed using ResFinder 4.0 [43], the plasmid replicon types were determined by PlasmidFinder [44], the virulence gene content was analysed by VirulenceFinder [45] and the serotypes of salmonella and *E. coli* were predicted by SeqSero 1.2. [46] and SeroTypeFinder 2.0. [47] at the Center of Genomic Epidemiology website (https://cge.cbs.dtu.dk/services/) (accessed on the 5 December 2021). The assembled contigs were also used to define the strains’ multi-locus sequence types (MLST) and core genome MLST types (cgMLST) using the Ridom SeqSphere+ software (GmbH, Münster, Germany). In *E. coli,* a cgMLST cluster was defined if having ≤10 non-identical target gene out of 2513. The sequence data generated were uploaded to the European Nucleotide Archives under project number PRJEB49171.

Pulsed field gel electrophoresis, using the CHEF Mapper (Bio-Rad Laboratories, Hercules, CA, USA), was performed for those isolates, which belonged to a species not having MSLT and cgMLST schemes [48].

### 4.5. Characterization of the mcr-Carrying Plasmids

Conjugation of the *mcr* plasmids from the wild type strains was attempted as previously described [14] using a rifampicin and Na-azide resistant recipient (*E. coli* J53_RAZ_). Selective plates contained 2 µg/mL of colistin and 100 µg/mL Na-azide. If conjugation attempts failed, or did not result in a derivative containing a single plasmid, only, attempts were made to transfer the *mcr* gene bearing plasmid by heat-shock transformation as follows: plasmids purified from the wild-type strain using the Qiagen Plasmid Maxi Prep (Qiagen, Hilden, Germany) were transformed into competent cells of *E. coli* DH5α as described [49] applying selective plates with 2 µg/mL colistin. Plasmid content, whether single or multiple, of transconjugants and transformants, was assessed by plasmid gel electrophoresis. Plasmids were purified from the single-plasmid-containing transconjugants or transformants by Qiagen Plasmid Maxi Prep kit Qiagen, Hilden, Germany) and their restriction fragment length polymorphism (RFLP) pattern was established. Aliquots of plasmids of approximately 60 kb were separately digested with 60 units of *Hinc*II, *Nsi*I, and 120 units of *Nde*I, while aliquots of other plasmids were digested with 120 units of *BamH*I, *Hind*III and *EcoR*I (New England Biolabs, Ipswich, MA, USA). In case no single plasmid derivative could be obtained, attempts were made to identify the location of the *mcr* gene by Southern blotting and hybridization of the Hybond N+ membrane-transferred plasmid gel patterns of the wild type isolates using a DIG DNA labelled probe (Roche, Mannheim, Germany), as described [50].

Purified plasmids representing each size and RFLP pattern combinations were sent for commercial sequencing on the Illumina MiSeq platform at the CCIB DNA Core Facility in Massachusetts General Hospital (Cambridge, MA, USA). The reads were assembled into contigs using CLC Genomic Workbench v20.0 (QIAGEN Aarhus, Denmark). Without assembling the complete circularised plasmids, these contigs were uploaded to the ResFinder and PlasmidFinder at the Center of Genomic Epidemiology (https://cge.cbs.dtu.dk/services/) (accessed on 5 December 2021) for identification of acquired antibiotic resistance genes co-located on the MCR plasmids, as well as to identify these plasmids’ incompatibility types.

### 4.6. Statistical Analysis

The frequency of virulence factor genes between groups was compared by Student’s *t* test.

## Figures and Tables

**Table 1:** Variants of wild type strains encountered.

Variant	Species	MLST	Resistance besides Colistin	MCR Plasmid			No. Present in Farm			
				Size (kb)	Inc Type	Antibiotic Resistance Co-Transferred	A	B	C	D
1	*E. coli*	ST48	AMP, NAL, CIP, SXT, TET, DOX	60	**IncI2**	None	2			
2	*E. coli*	ST69	AMP, NAL, CIP, TET, GM, TOB	60	*IncI2*	None		1		
3	*E. coli*	ST93	AMP, NAL, CIP, CHL, GM	60	*IncI2*	None			1	
4	*E. coli*	ST101	AMP, NAL, CIP, TET, DOX, CHL, (SXT), (GM)	60	**IncI2**	None		1		4
5	*E. coli*	CC162 *	AMP, NAL, CIP, TET, DOX, (SXT), (CHL)	60	**IncI2**	None	2		1	
6	*E. coli*	ST165	AMP, NAL, CIP, SXT, TET, DOX, GM, TOB	>150 ^&^	NA	NA				1
7	*E. coli*	ST354	AMP, NAL, CIP, SXT, TET, DOX, CHL, GM	>150	**IncHI2**	AMP, TET, DOX, CHL			1	3
8	*E. coli*	ST533	AMP, NAL, CIP, TET, DOX, CHL, GM, TOB	60	*IncI2*	None			1	
9	*E. coli*	ST1011	AMP, NAL, CIP, SXT, TET, DOX, CHL, GM, TOB	NA	NA	NA				1
10	*E. coli*	ST1140	AMP, NAL, CIP, SXT, TET, DOX, CHL, (AK), (GM), (TOB)	60	*IncI2*	None		2		
11	*E. coli*	ST1196	AMP, NAL, CIP, SXT, TET, DOX, CHL	35	**IncX4**	None		1		
12	*E. coli*	ST1196	AMP, NAL, CIP, SXT, TET, DOX, CHL	60	**IncI2**	None		1	1	
13	*E. coli*	ST1290	AMP, CPD, CTX, NAL, CIP, SXT, TET, DOX, CHL, FOS	60	*IncI2*	None	1			
14	*E. coli*	ST1485	AMP, NAL, CIP, SXT, TET, DOX, CHL	>150	**IncHI2**	AMP, TET, DOX, CHL			4	
15	*E. coli*	ST1585	AMP, CPD, NAL, CIP, TET, DOX, (CPD), (SXT), (GM)	60	**IncI2**	None			4	
16	*E. coli*	ST1630	AMP, NAL, SXT, TET, DOX, CHL, GM	60	**IncI2**	None				1
17	*E. albertii*	NA	AMP, NAL, TET, DOX, (CIP)	60	**IncI2**	None			2	
18	*K. pneumoniae*	ST340	AMP, CPD, CAZ, CTX, AZT, NAL, CIP, SXT, TET, DOX, CHL, GM	35	**IncX4**	None				2
19	*S. minnesota*	ST548	AMP, AMC, FOX, CPD, CAZ, CTX, CIP, SXT, TET, DOX, AK, GM, TOB	35	**IncX4**	None				1
**TOTAL NUMBER OF SPECIES/ST/PLASMID VARIANTS PER FARM**							**3**	**5**	**8**	**7**

* Including ST162 and its single locus variant (SLV):ST12220; Inc type—bold: determined from the plasmid sequence, italics: predicted from the Inc type of same size, same RFLP pattern type plasmids. ^&^ Molecular mass determined by Southern blot of the plasmid electrophoresis. Antibiotics in parentheses indicate that not all members of the same ST exhibited resistance to them. Abbreviations: AMP: ampicillin, AMC: amoxicillin-clavulanate, CPD: cefpodoxime, CAZ: ceftazidime, CTX: cefotaxime, FOX: cefoxitin, FOS: fosfomycin, NAL: nalidixic acid, CIP: ciprofloxacin, SXT: trimethoprim-sulphamethoxazole, TET: tetracycline, DOX: doxycycline, CHL: chloramphenicol, AK: amikacin, GM: gentamicin, TOB: tobramycin3.

## Data Availability

The bacterial whole-genome sequence data generated were uploaded to the European Nucleotide Archives under project number PRJEB49171.

## Supplementary Materials

The following are available online at https://www.mdpi.com/article/10.3390/antibiotics11030305/s1, Table S1: Characteristics of the mcr-positive wild type isolates and their single MCR-plasmid carrying derivatives. Table S2: Antibiotic resistance genes carried by *mcr-1* positive isolates, Table S3: Virulence genes carried by *mcr-1* positive isolates Table S4: WGS Assembly statistics.

## References

[1] Shen Y., Zhang R., Schwarz S., Wu C., Shen J., Walsh T.R., Wang Y. (2020). Farm animals and aquaculture: Significant reservoirs of mobile colistin resistance genes. Environ. Microbiol..

[2] Matamoros S., van Hattem J.M., Arcilla M.S., Willemse N., Melles D.C., Penders J., Vinh T.N., Hoa N.T., Bootsma M.C.J., van Genderen P.J. (2017). Global phylogenetic analysis of *Escherichia coli* and plasmids carrying the *mcr-1* gene indicates bacterial diversity but plasmid restriction. Sci. Rep..

[3] Bradford P.A., Kazmierczak K.M., Biedenbach D.J., Wise M.G., Hackel M., Sahm D.F. (2015). Correlation of beta-Lactamase Production and Colistin Resistance among Enterobacteriaceae Isolates from a Global Surveillance Program. Antimicrob. Agents Chemother..

[4] Poirel L., Jayol A., Nordmann P. (2017). Polymyxins: Antibacterial Activity, Susceptibility Testing, and Resistance Mechanisms Encoded by Plasmids or Chromosomes. Clin. Microbiol. Rev..

[5] Nang S.C., Li J., Velkov T. (2019). The rise and spread of mcr plasmid-mediated polymyxin resistance. Crit. Rev. Microbiol..

[6] Caniaux I., Van Belkum A., Zambardi G., Poirel L., Gros M.F. (2017). MCR: Modern colistin resistance. Eur. J. Clin. Microbiol..

[7] Zowawi H.M., Sartor A.L., Balkhy H.H., Walsh T.R., Al Johani S.M., Aljindan R.Y., Alfaresi M., Ibrahim E., Al-Jardani A., Al-Abri S. (2014). Molecular Characterization of Carbapenemase-Producing *Escherichia coli* and Klebsiella pneumoniae in the Countries of the Gulf Cooperation Council: Dominance of OXA-48 and NDM Producers. Antimicrob. Agents Chemother..

[8] Sonnevend A., Ghazawi A.A., Hashmey R., Jamal W., Rotimi V.O., Shibl A.M., Al-Jardani A., Al-Abri S.S., Tariq W.U.Z., Weber S. (2015). Characterization of Carbapenem-Resistant Enterobacteriaceae with High Rate of Autochthonous Transmission in the Arabian Peninsula. PLoS ONE.

[9] Moubareck C.A., Mouftah S.F., Pal T., Ghazawi A., Halat D.H., Nabi A., AlSharhan M.A., AlDeesi Z.O., Peters C.C., Celiloglu H. (2018). Clonal emergence of Klebsiella pneumoniae ST14 co-producing OXA-48-type and NDM carbapenemases with high rate of colistin resistance in Dubai, United Arab Emirates. Int. J. Antimicrob. Agents.

[10] Sonnevend Á., Ghazawi A., Darwish D., Barathan G., Hashmey R., Ashraf T., Rizvi T.A., Pál T. (2020). In vitro efficacy of ceftazidime-avibactam, aztreonam-avibactam and other rescue antibiotics against carbapenem-resistant Enterobacterales from the Arabian Peninsula. Int. J. Infect. Dis..

[11] Hala S., Antony C., Momin A., Alshehri M., Ben-Rached F., Al-Ahmadi G., Zakri S., Baadhaim M., Alsaedi A., Al Thaqafi O. (2021). Co-occurrence of *mcr-1* and *mcr-8* genes in multi-drug-resistant Klebsiella pneumoniae from a 2015 clinical isolate. Int. J. Antimicrob. Agents.

[12] Alghoribi M.F., Doumith M., Upton M., Al Johani S.M., Alzayer M., Woodford N., Ellington M.J., Balkhy H.H. (2019). Complete Genome Sequence of a Colistin-Resistant Uropathogenic *Escherichia coli* Sequence Type 131 fimH22 Strain Harboring *mcr-1* on an IncHI2 Plasmid, Isolated in Riyadh, Saudi Arabia. Microbiol. Resour. Announc..

[13] Forde B.M., Zowawi H.M., Harris P.N.A., Roberts L.W., Ibrahim E., Shaikh N., Deshmukh A., Ahmed M.S., Al Maslamani M., Cottrell K. (2018). Discovery of *mcr-1* -Mediated Colistin Resistance in a Highly Virulent *Escherichia coli* Lineage. Msphere.

[14] Sonnevend A., Ghazawi A., Alqahtani M., Shibl A., Jamal W., Hashmey R., Pal T. (2016). Plasmid-mediated colistin resistance in *Escherichia coli* from the Arabian Peninsula. Int. J. Infect. Dis..

[15] Tsui C.K.M., Sundararaju S., Al Mana H., Hasan M.R., Tang P., Perez-Lopez A. (2020). Draft Genome Sequence of an Extended-Spectrum beta-Lactamase-Producing Klebsiella oxytoca Strain Bearing *mcr-9* from Qatar. Microbiol. Resour. Announc..

[16] Tsui C.K.M., Sundararaju S., Mana H.A., Hasan M.R., Tang P., Perez-Lopez A. (2020). Plasmid-mediated colistin resistance encoded by *mcr-1* gene in *Escherichia coli* co-carrying blaCTX-M-15 and blaNDM-1 genes in pediatric patients in Qatar. J. Glob. Antimicrob. Resist..

[17] Eltai N.O., Kelly B., Al-Mana H.A., Ibrahim E.B., Yassine H.M., Al Thani A., Al Maslmani M., Lammens C., Xavier B.B., Malhotra-Kumar S. (2020). Identification of *mcr-8* in Clinical Isolates From Qatar and Evaluation of Their Antimicrobial Profiles. Front. Microbiol..

[18] Mohsin J., Pal T., Petersen J.E., Darwish D., Ghazawi A., Ashraf T., Sonnevend A. (2018). Plasmid-Mediated Colistin Resistance Gene *mcr-1* in an *Escherichia coli* ST10 Bloodstream Isolate in the Sultanate of Oman. Microb. Drug Resist..

[19] Hmede Z., Kassem I.I. (2018). The Colistin Resistance Gene *mcr-1* Is Prevalent in Commensal *Escherichia coli* Isolated from Preharvest Poultry in Lebanon. Antimicrob. Agents Chemother..

[20] Al-Mir H., Osman M., Drapeau A., Hamze M., Madec J.-Y., Haenni M. (2021). WGS Analysis of Clonal and Plasmidic Epidemiology of Colistin-Resistance Mediated by mcr Genes in the Poultry Sector in Lebanon. Front. Microbiol..

[21] Moawad A.A., Hotzel H., Neubauer H., Ehricht R., Monecke S., Tomaso H., Hafez H.M., Roesler U., El-Adawy H. (2018). Antimicrobial resistance in Enterobacteriaceae from healthy broilers in Egypt: Emergence of colistin-resistant and extended-spectrum beta-lactamase-producing Escherichia coli. Gut Pathog..

[22] Sadek M., de la Rosa J.O., Maky M.A., Dandrawy M.K., Nordmann P., Poirel L. (2021). Genomic Features of *MCR-1* and Extended-Spectrum β-Lactamase-Producing Enterobacterales from Retail Raw Chicken in Egypt. Microorganisms.

[23] Azam M., Mohsin M., Johnson T.J., Smith E.A., Johnson A., Umair M., Saleemi M.K. (2020). Genomic landscape of multi-drug resistant avian pathogenic *Escherichia coli* recovered from broilers. Vet. Microbiol..

[24] Javed H., Saleem S., Zafar A., Ghafoor A., Bin Shahzad A., Ejaz H., Junaid K., Jahan S. (2020). Emergence of plasmid-mediated mcr genes from Gram-negative bacteria at the human-animal interface. Gut Pathog..

[25] Eltai N.O., Abdfarag E.A., Al-Romaihi H., Wehedy E., Mahmoud M.H., Alawad O.K., Al-Hajri M.M., Al Thani A.A., Yassine H.M. (2017). Antibiotic Resistance Profile of Commensal *Escherichia coli* Isolated from Broiler Chickens in Qatar. J. Food Prot..

[26] Lima T., Domingues S., Da Silva G.J. (2019). Plasmid-Mediated Colistin Resistance in Salmonella enterica: A Review. Microorganisms.

[27] Campos J., Mourão J., Silveira L., Saraiva M., Correia C.B., Maçãs A.P., Peixe L., Antunes P. (2018). Imported poultry meat as a source of extended-spectrum cephalosporin-resistant CMY-2-producing Salmonella Heidelberg and Salmonella Minnesota in the European Union, 2014–2015. Int. J. Antimicrob. Agents.

[28] Li Q., Wang H., Xu Y., Bai X., Wang J., Zhang Z., Liu X., Miao Y., Zhang L., Li X. (2018). Multidrug-resistant Escherichia albertii: Co-occurrence of beta-Lactamase and *MCR-1* Encoding Genes. Front. Microbiol..

[29] Rodrigues A.C.S., Santots I.C.O., Campos C.C., Rezende I.N., Ferreira Y.M., Chaves C.E.V., Rocha-de-Souza C.M., Carvalho-Assef A.P.D.A., Chang M.R. (2019). Non-clonal occurrence of pmrB mutations associated with polymyxin resistance in carbapenem-resistant Klebsiella pneumoniae in Brazil. Mem. Inst. Oswaldo Cruz.

[30] Borges C.A., Tarlton N.J., Riley L.W. (2019). Escherichia coli from Commercial Broiler and Backyard Chickens Share Sequence Types, Antimicrobial Resistance Profiles, and Resistance Genes with Human Extraintestinal Pathogenic Escherichia coli. Foodborne Pathog. Dis..

[31] Bélanger L., Garenaux A., Harel J., Boulianne M., Nadeau E., Dozois C.M. (2011). Escherichia colifrom animal reservoirs as a potential source of human extraintestinal pathogenic, *E. coli.* FEMS Immunol. Med. Microbiol..

[32] Elmonir W., El-Aziz N.A., Tartor Y., Moustafa S., Remela E.A., Eissa R., Saad H., Tawab A. (2021). Emergence of Colistin and Carbapenem Resistance in Extended-Spectrum β-Lactamase Producing Klebsiella pneumoniae Isolated from Chickens and Humans in Egypt. Biology.

[33] Lupo A., Saras E., Madec J.-Y., Haenni M. (2018). Emergence of blaCTX-M-55 associated with fosA, rmtB and mcr gene variants in *Escherichia coli* from various animal species in France. J. Antimicrob. Chemother..

[34] Sun J., Fang L.-X., Wu Z., Deng H., Yang R.-S., Li X.-P., Li S.-M., Liao X.-P., Feng Y., Liu Y.-H. (2017). Genetic Analysis of the IncX4 Plasmids: Implications for a Unique Pattern in the *mcr-1* Acquisition. Sci. Rep..

[35] Hoang V.-T., Dao T.-L., Ly T.D.A., Gouriet F., Hadjadj L., Belhouchat K., Chaht K.L., Yezli S., Alotaibi B., Raoult D. (2021). Acquisition of multidrug-resistant bacteria and encoding genes among French pilgrims during the 2017 and 2018 Hajj. Eur. J. Clin. Microbiol..

[36] Leangapichart T., Gautret P., Brouqui P., Memish Z.A., Raoult D., Rolain J.M. (2016). Acquisition of *mcr-1* Plasmid-Mediated Colistin Resistance in *Escherichia coli* and Klebsiella pneumoniae during Hajj 2013 and 2014. Antimicrob. Agents Chemother..

[37] Alali W.Q., Thakur S., Berghaus R., Martin M.P., Gebreyes W.A. (2010). Prevalence and Distribution of Salmonella in Organic and Conventional Broiler Poultry Farms. Foodborne Pathog. Dis..

[38] Rebelo A.R., Bortolaia V., Kjeldgaard J.S., Pedersen S.K., Leekitcharoenphon P., Hansen I.M., Guerra B., Malorny B., Borowiak M., Hammerl J.A. (2018). Multiplex PCR for detection of plasmid-mediated colistin resistance determinants, *mcr-1*, *mcr-2*, *mcr-3*, *mcr-4* and *mcr-5* for surveillance purposes. Eurosurveillance.

[39] Borowiak M., Fischer J., Hammerl J.A., Hendriksen R.S., Szabo I., Malorny B. (2017). Identification of a novel transposon-associated phosphoethanolamine transferase gene, *mcr-5*, conferring colistin resistance in d-tartrate fermenting Salmonella enterica subsp. enterica serovar Paratyphi B. J. Antimicrob. Chemother..

[40] Watanabe K., Kodama Y., Harayama S. (2001). Design and evaluation of PCR primers to amplify bacterial 16S ribosomal DNA fragments used for community fingerprinting. J. Microbiol. Methods.

[41] Kado C.I., Liu S.T. (1981). Rapid procedure for detection and isolation of large and small plasmids. J. Bacteriol..

[42] Clinical Laboratory Standards Institute (2019). Performance Standards for Antimicrobial Susceptibility Testing.

[43] Zankari E., Hasman H., Cosentino S., Vestergaard M., Rasmussen S., Lund O., Aarestrup F.M., Larsen M.V. (2012). Identification of acquired antimicrobial resistance genes. J. Antimicrob. Chemother..

[44] Carattoli A., Zankari E., Garcia-Fernandez A., Voldby Larsen M., Lund O., Villa L., Aarestrup F.M., Hasman H. (2014). PlasmidFinder and pMLST: In silico detection and typing of plasmids. Antimicrob. Agents Chemother..

[45] Tetzschner A.M.M., Johnson J.R., Johnston B.D., Lund O., Scheutz F. (2020). In Silico Genotyping of *Escherichia coli* Isolates for Extraintestinal Virulence Genes by Use of Whole-Genome Sequencing Data. J. Clin. Microbiol..

[46] Zhang S., Yin Y., Jones M.B., Zhang Z., Kaiser B.L.D., Dinsmore B.A., Fitzgerald C., Fields P.I., Deng X. (2015). Salmonella Serotype Determination Utilizing High-Throughput Genome Sequencing Data. J. Clin. Microbiol..

[47] Joensen K.G., Tetzschner A.M., Iguchi A., Aarestrup F.M., Scheutz F. (2015). Rapid and easy in silico serotyping of *Escherichia coli* using whole genome sequencing (WGS) data. J. Clin. Microbiol..

[48] Gautom R.K. (1997). Rapid pulsed-field gel electrophoresis protocol for typing of *Escherichia coli* O157:H7 and other gram-negative organisms in 1 day. J. Clin. Microbiol..

[49] Pál T., Ghazawi A., Darwish D., Villa L., Carattoli A., Hashmey R., Aldeesi Z., Jamal W., Rotimi V., Al-Jardani A. (2017). Characterization of NDM-7 Carbapenemase-Producing *Escherichia coli* Isolates in the Arabian Peninsula. Microb. Drug Resist..

[50] Mouftah S.F., Pal T., Darwish D., Ghazawi A., Villa L., Carattoli A., Sonnevend Á. (2019). Epidemic IncX3 plasmids spreading carbapenemase genes in the United Arab Emirates and worldwide. Infect. Drug Resist..

